# Effects of Essential Oils from *Eucalyptus globulus* Leaves on Soil Organisms Involved in Leaf Degradation

**DOI:** 10.1371/journal.pone.0061233

**Published:** 2013-04-05

**Authors:** Carla Martins, Tiago Natal-da-Luz, José Paulo Sousa, Maria José Gonçalves, Lígia Salgueiro, Cristina Canhoto

**Affiliations:** 1 Institute of Marine Research (IMAR-CMA), Department of Life Sciences, University of Coimbra, Coimbra, Portugal; 2 Center for Pharmaceutical Studies (CEF), Faculty of Pharmacy, University of Coimbra, Coimbra, Portugal; Roehampton university, United Kingdom

## Abstract

The replacement of native Portuguese forests by *Eucalyptus globulus* is often associated with deleterious effects on terrestrial and aquatic communities. Several studies have suggested that such a phenomenon is linked with the leaf essential oils released into the environment during the Eucalyptus leaf degradation process. However, to date, the way these compounds affect leaf degradation in terrestrial systems i.e. by direct toxic effects to soil invertebrates or indirectly by affecting food of soil fauna, is still unknown. In order to explore this question, the effect of essential oils extracted from *E. globulus* leaves on terrestrial systems was investigated. Fungal growth tests with species known as leaf colonizers (*Mucor hiemalis*, *Alternaria alternata*, *Penicillium* sp., *Penicillium glabrum* and *Fusarium roseum*) were performed to evaluate the antifungal effect of essential oils. In addition, a reproduction test with the collembolans *Folsomia candida* was done using a gradient of eucalyptus essential oils in artificial soil. The influence of essential oils on feeding behaviour of *F. candida* and the isopods *Porcellio dilatatus* was also investigated through food avoidance and consumption tests. Eucalyptus essential oils were lethal at concentrations between 2.5–20 µL/mL and inhibited growth of all fungal species between 1.25–5 µL/mL. The collembolan reproduction EC_50_ value was 35.0 (28.6–41.2) mg/kg and both collembola and isopods preferred leaves without oils. Results suggested that the effect of essential oils in leaf processing is related to direct toxic effects on fungi and soil fauna and to indirect effects on the quality and availability of food to soil invertebrates.

## Introduction

During the last decades, the Portuguese native forests have been replaced by *Eucalyptus* spp. plantations [1]. Monospecific stands of *Eucalyptus globulus* Labill. (Myrtaceae) represent approximately 23% of the forested area of the country [2]. A large number of studies have suggested that these replacements induce losses in the diversity of the edaphic fauna of Portuguese forests [3]–[5], comprising losses of rare and endemic invertebrates (e.g. the collembola species *Stenognathellus denisi* and *Folsomides navacerradensis*) and the emergence of opportunistic species with high reproduction rates (e.g. the collembola species *Xenilla brevisimilis mediterranea* and *Folsomia sexoculata*; [4]–[8]). Such changes have been associated with a characteristic soil hydrophobicity, reduced hydraulic conductivity [9]–[11] and presence of allelopathic compounds [12], [13], all contributing to a simplification of the habitat and the creation of essentially a monospecific forest [10], [14], [15]. Other studies have suggested that the reduced diversity of soil fauna in these forests is related to low nutrient content of the leaf litter and high concentrations of secondary compounds like essential oils and phenolic compounds [14], [16]. After senescence, the leaf cuticle loses its integrity, leaf leaching is facilitated and secondary compounds, such as polyphenols and essential oils, may accumulate in the upper layers of the soil, influencing soil chemical characteristics and nutrient content [17], [18]. Although soluble phenolic compounds and tannins, may have a short life-time in the soils [19], the effect of the oils maybe extended due to their hydrophobicity and oil vesicles prolonged integrity. Eucalyptus oils are known to inhibit or suppress microbial and fungal growth [20], [21], reducing consumption by soil invertebrates, leaf digestion and survival of aquatic and terrestrial invertebrates [20], [22]–[24]. Given the role of fungi and bacteria in increasing the palatability of leaves to consumers [25], [26], [27], these recent findings could also explain an indirect influence of these compounds on soil invertebrates (namely detritivores and fungivores).

Collembola and isopods are soil invertebrates that play a crucial role in litter decomposition [28], [29], [30]. Collembolans are the most abundant arthropods after mites [31]. Their role in decomposition is mainly due to their grazing activity on fungal hyphae, which enhances fungal growth [32], [33] and consequently may accelerate the decomposition rate of leaves. Terrestrial isopods affect litter decomposition mainly by the fragmentation of leaves and by influencing microbial dynamics through alteration of the substrate quality, when the vegetal material is excreted as faeces. They contribute to an increase in substrate surface area accessible to microbial attack and to an increase of substrate pore volume and aeration, thus enhancing the overall microbial resource exploitation [34], [35]. Some studies have shown effects of essential oils on key-species of collembola (by evaluating fumigant toxicity; [36]) and other terrestrial invertebrates (by evaluating repellent activity; [37]). Despite these studies, to date, the route of toxicity is still unknown, i.e. direct repellence, contact, fumigation, and digestive mediated toxic effects or indirectly by decreasing the nutrient status of the detritus. The effect of essential oils from *E. globulus* leaves on the reproduction of the collembolan, *Folsomia candida*, and on the food preference of *F. candida* and the isopods *Porcellio dilatatus* was investigated in this study. In addition, the antifungal activity of the eucalyptus essential oils was evaluated, using terrestrial fungal species commonly found in decomposing litter.

## Materials and Methods

### Extraction of essential oil

Adult fresh *Eucalyptus globulus* leaves were collected from one adult tree in Castelo Branco City, Portugal (N 39° 54′, W 7° 37′), and taken to the laboratory in plastic containers for the essential oil extraction. The essential oil was isolated by hydrodistillation using a modified Clevenger device as described in the European Pharmacopeia [38]. Distilled water was mixed with the *E. globulus* leaves (previously cut into pieces less than 2×2 cm) on the day after collection, in a proportion of 1∶10 (w/v, leaves∶water), and the distillation was performed for 2.5 h with a flow of 3 mL/min. After boiling, condensing and decantation, the obtained oil was stored in hermetic bottles at 4°C. The yield of oil per leaf tissue (in v/w) was determined according to European Pharmacopoeia [38]. No specific permits were required for leaf collection because this was done at a public location from a non-privately-owned tree and the procedures adopted did not considerably affect the health of the tree. Moreover *E. globulus* is an exotic tree species in Portugal and is not protected or endangered.

### Fungus growth test

The effect of eucalyptus essential oils on fungal growth was investigated using 5 species of fungi, commonly found in decomposing litter of soil systems [44], as test organisms: *Mucor hiemalis* Wehmer, *Alternaria alternata* (Fr.) Keissl., *Penicillium* sp., *Penicillium glabrum* (Wehmer) Westling and *Fusarium roseum* (Link) Snyd.. Fungi were obtained from laboratory cultures and kept in Sabouraud broth. Prior to testing, each species was inoculated in Sabouraud agar to ensure optimal growth rates and purity. A macrodilution broth method was used to determine the minimal inhibitory concentrations of the oil (MICs) according to the Clinical and Laboratory Standards Institute (CLSI) reference protocols M38-A2 [45], for filamentous fungi. Serial two-fold dilutions of the oil were prepared in dimethyl sulfoxide (DMSO) in order to obtain test concentrations ranging from 0.64 to 20.0 µL/mL. Final concentrations of DMSO never exceeded 2% v/v. Recent cultures of each strain were used to prepare the cell suspension adjusted to 1−2×104 cells per mL in RPMI 1640 broth (with L-glutamine, without bicarbonate, and the pH indicator phenol red) and distributed into 12×75 mm glass test tubes. The concentration of cells was confirmed by viable count on Sabouraud agar. Oil-free growth controls and DMSO control tubes, were also included. The test tubes were incubated aerobically at 35°C for 48 h. MIC values were determined as the lowest concentration of the oil causing full growth inhibition. To measure minimal lethal concentrations (MLCs), 20 µl samples were taken from each negative tube, plus the first tube showing growth (to serve as a growth control) after MIC reading to SDA plates and incubated at 35°C for 48 h. MLC values were determined as the lowest concentration of the oil causing fungal death. All experiments were performed in triplicate and repeated whenever the results of each triplicate did not agree. This methodology is widely used for medicinal purposed [46], [47] and has been used for the antifungal evaluation of several essential oils [48]–[51].

### Soil invertebrate species

The collembolan *Folsomia candida* Willem (Colembolla: Isotomidae), and the isopod *Porcellio dilatatus* Brandt (Isopoda: Porcellionidae) were used as test organisms. These species are representative of soil decomposers and have been used in ecotoxicological tests (e.g. [39], [40]) due to their sensitivity to the presence of contaminants in soil. *F. candida* is recommended as a test species in ISO guidelines for laboratory ecotoxicological tests [41], [42] and recently was used in laboratory tests to evaluate interactions with fungal communities [43].

Collembolans were obtained from laboratory cultures at Coimbra University and maintained at 20±2°C, under a photoperiod of 16∶8 h light∶ dark. A mixture of plaster of Paris and activated charcoal in a ratio of 11∶1 (w∶w) was used as culture substrate in cylindrical transparent plastic boxes (11 cm diameter and 4 cm height). Granulated dry yeast was added as food in small amounts to avoid spoilage by fungi. When detected, mouldy food was removed from the culture containers. In ecotoxicological tests, collembolans of 10 to 12 days old obtained from synchronized cultures, were used [41].

Isopods were collected in a field in the village of S. Domingos from South of Portugal, and maintained in the laboratory in an acclimatized room at 20±2 °C under a photoperiod of 16∶8 h light∶dark, using a mixture of horse dung and potting soil (both previously defaunated through two freeze–thawing cycles of 48 h at −20°C followed by 48 h at 25°C) as substrate. Oven-dried alder leaves (at 40°C for 48 h) and potato fragments were added as food. Only adult isopods were used in the ecotoxicological tests, independently of their sex, although pregnant females were not used in the experiments. No specific permits were required for Isopods collection because this was done at a public location and *P. dilatatus* is not a protected or an endangered species. Moreover, the number of specimens collected did not cause significant decline in the local population.

### Collembolan reproduction test

Artificial soil composed by 10% of *Sphagnum* peat, 20% of kaolinite clay, and 70% of industrial quartz sand with the pH adjusted to 6.0±0.5 through the addition of calcium carbonate was used. Treatments consisted of artificial soil spiked with eucalyptus essential oil in the laboratory. For this, gradients of solutions with increasing oil concentrations were prepared in water. Although the eucalyptus essential oil was not miscible with water (due to its hydrophobicity), these constituents were shaken thoroughly for three minutes by hand to obtain mixtures as homogeneous as possible immediately before being added to the soil. Each mixture was added to a portion of artificial soil to achieve the desired concentrations and to reach 50% of the soil water-holding capacity at the moment before the beginning of the experiment. Collembolan tests were performed under the same temperature and photoperiod of the laboratory cultures.

#### Survival test

To define the range of oil concentrations that should be tested in the collembolan reproduction test, a preliminary range-finding test was performed as described by ISO [41]. A gradient of six exposure concentrations was used: 0, 1, 10, 100, 1000, and 10000 mg of oil/kg of soil (dry weight; DW). Five replicates per test concentration were prepared, each consisting of glass containers (4 cm Ø and 7 cm height) filled with 30 g (DW). Ten springtails were placed into each test container and 2 mg of granulated dry yeast were added as food. The containers were closed and left for 14 days. The test containers were opened for few seconds twice a week to allow aeration. At the end of the test period, each test container was emptied into a small vessel filled with water. After the addition of few drops of blue ink and gently stirring, the surviving animals floating on the water surface were counted and the percentage of mortality was determined for each test concentration. Due to the rapid degradation of dead springtails, missing animals were assumed to have died during the test period.

#### Reproduction tests

As in the survival test, 100% of mortality was found in the test concentrations higher or equal to 100 mg/kg, the range of concentrations prepared for the reproduction tests was 0, 1, 3, 8, 22 and 60 mg/kg DW. This test followed the procedures described by ISO [41]. The methodology adopted was similar to that used in the survival test except the incubation period was 28 days and the addition of 2 mg of granulated dry yeast was done also after 14 days of incubation in each replicate. An additional replicate but without springtails was prepared for each concentration to determine the pH and soil moisture of the treatments at the end of the experiment. Extraction was the same as for the survival test. The animals floating on the water surface were photographed (using a digital camera) and counted using the Image Tool software (http://ddsdx.uthscsa.edu/dig/itdesc.html). Missing adult springtails were considered dead.

### Feeding behaviour

#### Test with collembolans

A food avoidance test with *F. candida* was done in cylindrical plastic containers (7 cm diameter and 6 cm height) with the bottom filled with a 1-cm layer of a mixture of plaster of Paris and activated charcoal in a ratio of 11∶1 (w∶w). A dividing line was drawn on the bottom of each replicate and, in each section, 20 mg of granulated yeast was added. In five of the test containers, the granulated yeast of both sections was moistened by adding 1.2 µL of water on top of it. These containers constituted the dual-control combination that was performed to demonstrate that in the absence of oil, there is a random food avoidance of the organisms in both sections of the test vessels. In the other five test containers, while the granulated yeast of one section was moistened with water (as performed in each section of the dual-control vessels), the yeast in the other section was impregnated with 1.2 µL of eucalyptus essential oil to reach a concentration of 0.06 µL of oil/mg of yeast DW (a realistic concentration for eucalyptus adult leaves according to Canhoto and Graça [20]). These last containers constituted the oil-control combination. Twenty springtails were introduced in the middle line of each test container and then each container was covered with a transparent lid. The test was performed at 20±2°C under constant light. The number of springtails in each section of the replicates was recorded every 30 min for 6 hours.

#### Tests with isopods

Feeding behaviour of *P. dilatatus* was investigated in two sets of tests. Firstly, a food avoidance test using similar conditions to the collembolan food avoidance test except a piece of alder leaf disc-shaped (Ø 1.2 cm) was provided in each section of each container instead of yeast, and fixed with a pin. The impregnation of the leaf discs with essential oil was performed using a solution of oil and ether to facilitate leaves homogeneous impregnation with oils. Previous tests indicated that the leaf impregnation with ether does not affect leaf consumption by isopods (possibly due to its fast evaporation after leaf impregnation). Ten replicates of a dual-control combination were prepared using alder-leaf discs moistened with ether only (1.1 µL) in both sections. The same number of replicates of an oil-control combination were prepared using alder-leaf discs moistened with ether (1.1 µL) in one section (control), and alder-leaf discs impregnated with 1.1 µL of 1∶1 essential oil∶ether (v∶v) solution in the other section to achieve a concentration of 0.06 µL of oil per mg of eucalyptus leaf DW (usual concentration in eucalyptus leaves according to Canhoto and Graça [20]). Ether was always allowed to evaporate for ten seconds after being added to the leaves in replicates of both dual-control and oil-control combinations. After evaporation, one *P. dilatatus* was introduced in the middle line of five test containers of each test combination (both dual-control and oil-control combinations), and five *P. dilatatus* were introduced in the middle line of other five test containers of each test combination. This procedure provided information on the feeding preference of the isopods when alone or in a group, since the food avoidance results with several isopods could be influenced by the release of a pheromone that stimulates their aggregation [52]. The experiment was done at 20±2°C, under constant light and the number of isopods in each section of the containers was recorded every 30 min for 6 hours.

The second set of tests aimed to investigate the feeding behaviour of the isopods through a food consumption test. Experimental setup was the same as the previous test. For each section of the test vessels, a pair of alder-leaf discs (Ø 1.2 cm) was cut from the same leaf, one of which was oven dried (at 40°C for 48 h) to determine its initial weight and the other was introduced in the bottom of the test vessel and fixed with a pin. The essential oil was diluted in ether and added to the leaf discs. In four containers, an alder-leaf disc was impregnated with 1.1 µL of 1∶1 essential oil∶ether solution (v∶v) to reach a concentration of 0.06 µL of oil/mg of leaf and placed in one of the sections, and an alder-leaf disc impregnated with the same volume of ether was placed in the other section (an oil-control combination). After ten seconds (to allow ether volatilization), three previously weighed individuals of *P. dilatatus* were introduced into each test container for 6 hours. After that period, the isopods were removed and the alder-leaf discs were oven dried (at 40°C for 48 h) and weighed. The leaf consumption was estimated by the difference between the initial and the final dry weight of the alder-leaf discs and expressed as mg dry leaf/mg animal. Four additional containers were prepared with an alder-leaf disc moistened with 1.1 µL of ether in each section as controls.

### Statistical analyses

Data obtained in the collembolan reproduction tests were analysed using STATISTICA version 7. Mortality and reproduction were analysed using one-way ANOVA followed by Dunnett's post hoc test, to calculate significant differences. The EC_50_ value (oil concentration that causes 50% reduction in reproduction) was calculated using the exponential regression model [53].

Data obtained in the food avoidance tests (except for tests with one isopod per test container) were analysed using Fisher exact test [54] for each observation time. This was performed to evaluate if a significant avoidance response occurred at each observation time. For the dual-control combinations, a two-tailed distribution was used, and for the oil-control combinations, a one-tailed test was done. The rejection of the null hypothesis occurred for a probability lower than or equal to 0.05. Before running the statistical test, the number of individuals found in each section of the replicates was corrected taking into account the mortality observed (dead organisms were equally divided by both sections of the containers). Data from the food avoidance test, using one isopod per test container, were analysed by the Chi-square test [54] for each time interval.

To evaluate if the avoidance behaviour changed over time the responses within the oil-control combination were compared to the mean number of organisms found in the control section at each observation (each 30 min) by a repeated measures ANOVA. A Duncan's post hoc test was applied when a significant variation on the behaviours among time was found. In the food consumption test with isopods, the differences between leaf consumptions of each section of the test containers, in the oil-control combination, were analysed by paired *t*-test [54]. The normality and homogeneity of data was always checked before ANOVAs, *t*-test and non-linear estimations. Since these assumptions were met, data were not transformed. Regarding fungal data, since the test protocol does not allow variation of data among replicates within each oil concentration tested, (full agreement of results between replicates was required to validate the test), no statistical analysis was needed to support conclusions from the data.

## Results

### Fungal growth test

All fungal species were moderately affected by the eucalyptus essential oil. Growth of the test species was inhibited for oil concentrations between 1.25 and 5 µL/mL and mortality was caused between 2.5 and 20 µL/mL. The test species of the genera *Penicillium* were least sensitive to the presence of essential oils compared to the other test fungal species; while growth of *M. hiemalis* was most sensitive and *A. alternata* and *F. roseum* species exhibited mortality at the lowest concentration (Table 1).

**Table 1:** Minimal inhibition concentration (MIC) and minimal lethal concentration (MLC) of *E. globulus* essential oils to fungi species.

Test species	MIC	MLC
*Alternaria alternata*	2.5	2.5
*Fusarium roseum*	2.5	2.5
*Mucor hiemalis*	1.25	10
*Penicillium glabrum*	5	20
*Penicillium* sp.	5	20

Concentrations were determined in a fungi growth test by the micro-dilution method and results were obtained from 3 independent experiments. Values are expressed in µL of oil/mL of agar.

### Collembolan reproduction test

**Figure 1:**
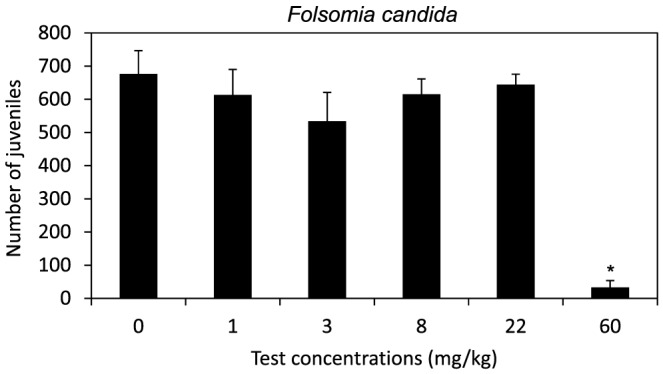
Reproduction test with *Folsomia candida*. Reproduction (mean ± standard deviation; *n* = 5) when exposed to soils spiked with increasing concentrations of essential oils of *Eucalyptus globulus* leaf in artificial soil. * indicates statistical differences compared to the control.

The validity criteria for collembolan reproduction test (≥30 juveniles/test container, mortality <20% and coefficient of variation <30% in control vessels) were fulfilled. No adult mortality was found at any of the test concentrations, except at the highest concentration (60 mg/kg), where 76% mortality was observed. Collembolan reproduction was significantly lower than controls (one-way ANOVA, F_(5,24)_ = 81.2, *p*<0.001) only at the highest test concentration (60 mg/kg; Figure 1). An EC_50_ value of 35.0 mg/kg (28.6–41.2 of 95% confidence limits) was estimated for eucalyptus essential oil.

### Feeding behaviour

#### Test with collembolans

**Figure 2:**
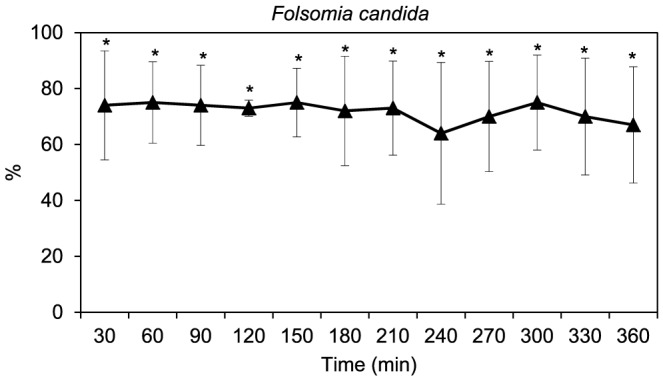
Food avoidance test with *Folsomia candida*. Percentage of individuals (mean ± standard deviation, *n* = 5) in the control section of containers combining yeast impregnated with eucalyptus essential oil (test section) and yeast without essential oil (control section) in the different observations performed over time. * indicates significantly (*p*≤0.05) higher percentage of organisms in the control section (with uncontaminated food) than in the section with oil-impregnated food after Fisher exact test.

The food avoidance test with *F. candida* showed a random distribution in the containers of the dual-control combination over time (Fisher exact test, *p*>0.05). The exception was after 180 minutes of exposure (Fisher exact test, *p* = 0.0062). In the oil-control combination, the number of organisms found in the control section was always significantly higher compared to that in the section with yeast impregnated with oil (Fisher exact test, *p*≤0.05) in each observation time (Figure 2). No significant differences were found between the number of organisms in the control section of the replicates with the oil-control combination over time (repeated measures ANOVA, F_(11,44)_ = 0.305, *p* = 0.981).

#### Tests with isopods

**Figure 3:**
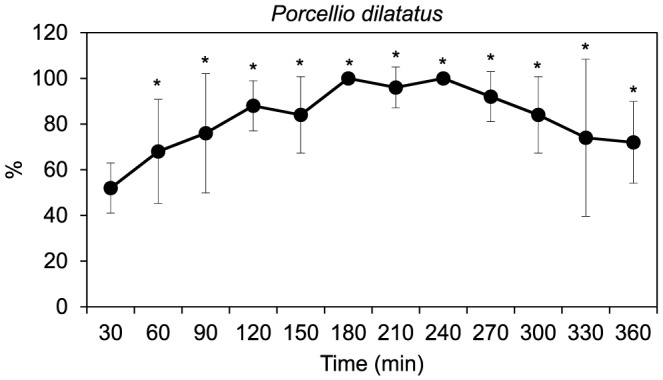
Food avoidance test with *Porcellio dilatatus*. Percentage of individuals (mean ± standard deviation, *n* = 5) in the control section of containers combining alder-leaf discs impregnated with eucalyptus essential oil (test section) and alder-leaf discs without essential oil (control section) in the different observations performed over time. * indicates significantly (*p*≤0.05) higher percentage of organisms in the control section (with uncontaminated food) than in the section with oil-impregnated food after Fisher exact test.

In the food avoidance tests with *P. dilatatus* (first set of tests) the dual-control combination showed homogeneous distribution along the two sections of the replicates over time independently of the number of individuals (whether one or five organisms) introduced in each test container at the beginning of the test (*X^2^*, *p*>0.05 and Fisher exact test, *p*>0.05 for one and five individuals per replicate, respectively). In the oil-control combination, the isopods generally showed a preference for the section with non-impregnated leaves (Fisher exact test, *p*≤0.05) over time (except in the first observation, T30) but only in containers with five organisms (Figure 3). This food avoidance behaviour significantly differed over time (repeated measures ANOVA, F_(11,44)_ = 3.533, *p* = 0.001). Significant differences were found between the first observation time (T30) and those from T120 till T300 and between the observation time T60 and those of T180 till T240, with the number of organisms found in the section with non-contaminated food gradually increasing over the first observation times. In the same oil-control combination, but using only one isopod per test container, no food avoidance behaviour was detected, i.e. there was a random distribution of the organisms along both sections of the containers (*X^2^*, *p*>0.05; data not shown).

The food consumption test with isopods (second set of tests) showed that in the dual-control combination no significant differences between the consumption of the alder-leaf discs from both sections (*t*-test, t_(6)_ = 0.183, *p* = 0.861) were found (data not shown). In the oil-control combinations, the addition of oils to alder leaves significantly decreased food consumption (*t*-test, t_(6)_ = 4.30, *p* = 0.005). The impregnation of alder leaf disks with oils decreased leaf consumption by about 72% (0.5±0.6 µg alder leaf-discs with oils DW/mg of isopod *vs*. 1.8±1.9 µg alder leaf-discs without oils DW/mg of isopod; average ± standard deviation, *n* = 4) after 6 hours of exposure.

## Discussion

The results obtained in the present study suggest that eucalyptus essential oils may influence the distribution and fitness of soil invertebrates either by direct toxicity or by reducing palatable food availability and quality. This could lead to consequences for soil functions (litter decomposition) related to key ecosystem services like nutrient cycling.

The effect of eucalyptus essential oils on fungi was species specific. These secondary compounds depressed fungal growth and limited fungal viability generally at relatively low concentrations (1.25–5 µL/mL and 2.5–20 µL/mL, respectively). These findings are in agreement with results from previous investigations performed with aquatic fungal species (aquatic Hyphomycetes [20], [55]). These studies showed that eucalyptus essential oils are able to differentially suppress or inhibit fungal growth even at a distance, due to oil vapours [20], and to affect their enzymatic (and though degradative) ability and viability [55]. Although such effects may be mitigated in aquatic environments (e.g. stream), due to an elongated integrity of the oil vesicles enveloped in the leaf cuticle and/or by water flow entrainment, the same may not occur in terrestrial environments where leaves accumulate and degradation is spatially more constrained. Such impairment of fungal growth may not only affect microbial decomposition patterns and rates but may also limit invertebrate comminution performance [43] and, consequently, nutrient recycling and secondary production. As fungi are important decomposer agents, leaf degradation may be directly affected by the inhibition of fungal colonization and/or by a decrease of leaves palatability to the invertebrates. We hypothesize that such an effect may be particularly important in invertebrates that feed on colonizing fungi rather than organisms that consume the leaf itself [56], [57]. Furthermore, this may be particularly relevant if the invertebrates present specific preferences for particular fungal species (e.g. [32], [33], [58]) as the collembolans *F. candida* [59]. For instance, in the present study, *A. alternata*, a common preferred species consumed by collembolan feeders [60], [61], was inhibited at the lowest test concentration of oils (2.5 µL/mL). On the other hand, both species of the genera *Penicillium*, the least affected in the fungal growth test, are often rejected by springtails as food [60]. Therefore, even though there is no consensus in the scientific community on the importance of fungal diversity on the process of leaf decomposition and their potential functional redundancy [62], [63], it seems possible that species specific effects of the eucalyptus oils on fungal growth may influence leaf decomposition through invertebrates feeding behaviour either by decreasing the quantity of available mycelial biomass or by interfering with the palatability of food items. This may indicate that important bottom-up (secondary production) and top-down (leaves decomposition and nutrient availability) effects can be expected.

In the present work, eucalyptus oils affected both collembolan reproduction and food preference of Collembola and isopods. The survival test performed with *F. candida* showed 100% mortality at concentrations of eucalyptus essential oils ≥100 mg/kg, and the reproduction test defined an EC_50_ value of 35.0 mg/kg. Considering essential oils have a high evaporation rate (non-published personal data demonstrated that 70 to 75% of the oil evaporates in the first 14 days after its impregnation in soil), and knowing that the test vessels were frequently aerated over the test period, the toxicity found in the collembola reproduction test is highly relevant. Moreover, these data are in agreement with the toxicity of eucalyptus essential oils in the Coleoptera, *Sitophilus oryzae* and *Acanthoscelides obtectus*, and Diptera, *Culex pipiens* [64]–[67]. Such toxicity is most probably related to the volatile components of the essential oils and, particularly in the case of collembolans, the formation of an impermeable film enveloping the body and, consequently, inhibiting breathing of the organisms. It may also be due to the presence of amphiphilic compounds (substances with both hydrophilic and lipophilic properties) that can penetrate in the cells of soil invertebrates, affecting their physiology [68].

Both invertebrate species (collembola and isopods) avoided food with eucalyptus oils, which is in agreement with previous findings that have shown that essential oils induce repulsive effects on invertebrate species [65], [66]. Observed behaviour of *F. candida* suggested that these test organisms strongly avoid food containing essential oils, at all observation times during the six hours immediately after its application. The feeding behaviour investigated in the two sets of tests with *P. dilatatus* (both food avoidance and food consumption tests) generally suggested a higher preference of isopods for food free of eucalyptus essential oil as observed for collembola. Although that preference of isopods was not observed at the individual level (one isopod per test container), this behaviour was clear when using groups of five individuals per test container. Most probably, such difference is related with the presence of an aggregation pheromone released by the organisms that might affect their behaviour [29]. In contrast to the response of collembola (for which the food avoidance behaviour did not significantly change over the exposure time), the distribution of isopods in the food avoidance tests, using five organisms per test container, suggested that the repellence of essential oil was significantly more effective two hours after oil impregnation in the alder-leaf discs. This fact could be explained by an increasing volatilization of the essential oil after that period (two hours) since, according to Regnault-Roger and Hamraoui [65], these compounds are generally more active when volatilizing. However, more data on the volatilization rate of eucalyptus essential oil over shorter time periods (hours) and under different environmental conditions would be needed to confirm this assumption.

In natural conditions, there is a constant supply of litter to the ground (due to eucalyptus leaf fall) and previous studies [23] have indicated that in an adult non cleaned eucalyptus forest 894 leaves (±246 standard error) per m^2^ can be found in the soil. Considering that an *E. globulus* leaf may contain up to 5% of its wet weight of essential oils, the input of essential oils to the soil is constant and potentially high (at least compared to the oil concentrations in the laboratory tests). The results obtained in the present work and such continuous supply may help to explain the observed changes in invertebrate assemblages of ecosystems affected by eucalyptus afforestation. Data obtained in this study also indicates that the direct application of eucalyptus essential oils in a terrestrial system may provoke immediate and short-term deleterious effects on edaphic fauna, which apparently supports the suitability of the use of these secondary compounds as bio-pesticide in the field [37].

## Conclusions

Results suggest that the essential oils released during eucalyptus leaf degradation may affect fungal growth, with important consequences on leaf microbial and invertebrate-mediated degradation, secondary production and on the provision of key-ecological services (e.g. nutrient cycling). The observed direct toxicity towards Collembola and isopods also suggests that these secondary compounds may constitute one of the main causes of the deleterious effects of eucalyptus afforestation on the biodiversity of native soil organisms. Further studies are needed, using species from other functional groups (e.g. representative of different routes of exposure) and species with different feeding traits to clearly understand the potential effect of oils on terrestrial systems.
